# Fungal Community and Biodeterioration Analysis of Hull Wood and Its Storage Environment of the Nanhai No. 1 Shipwreck

**DOI:** 10.3389/fmicb.2020.609475

**Published:** 2021-01-15

**Authors:** Yeqing Han, Xinduo Huang, Yu Wang, Jing Du, Kaixuan Ma, Yue Chen, Naisheng Li, Zhiguo Zhang, Jiao Pan

**Affiliations:** ^1^Ministry of Education Key Laboratory of Molecular Microbiology and Technology, Department of Microbiology, College of Life Sciences, Nankai University, Tianjin, China; ^2^Chinese Academy of Cultural Heritage, Beijing, China; ^3^National Center of Archaeology, Beijing, China

**Keywords:** Nanhai No 1 shipwreck, biodeterioration, fungal community, high-throughput sequencing, *Fusarium solani*, *Scedosporium apiospermum*

## Abstract

The Nanhai No. 1 shipwreck is a Chinese merchant ship in the Southern Song Dynasty, and now it is stored in a huge enclosed glass warehouse in Maritime Silk Road Museum in Guangdong Province. At present, the hull of the Nanhai No. 1 shipwreck is still being excavated, and a small part of the hull wood is soaked in a specific solution to desalt. Through long-term exploration, we found that the above two states of hull wood had undergone biodeterioration, so the purpose of this study is to analyze the fungal community of exposed and soaked wood from the Nanhai No. 1 shipwreck. We sampled 10 exposed hull wood and sea mud samples, two wood storage water samples, and air samples in the glass warehouse. We used scanning electron microscope and optical microscope to find that there were obvious fungal structures in exposed wood and wood storing water samples. High-throughput sequencing of fungi revealed that the most abundant genera in exposed and soaked wood were *Fusarium* sp., and *Scedosporium* sp., respectively. In addition, *Fusarium solani* and *Scedosporium apiospermum* were successfully isolated from the hull wood surface and wood storing water samples, and the degradation tests of lignin and cellulose, the sensitivity tests of biocides and growth curve assay were carried out. We also found that *Penicillium* sp. and *Cladosporium* sp. are the most abundant in the glass warehouse air. Our research results show that *F. solani* and *S. apiospermum* should be regarded as a major threat to the preservation of the Nanhai No. 1 shipwreck. These results provide a reference for our protection of shipwrecks and other similar artifacts.

## Introduction

In 1987, a Chinese shipwreck in the Southern Song Dynasty was found on the southwest coast of Guangdong Province, which was named Nanhai No. 1 shipwreck (Yong, 2019). In 2007, The Nanhai No. 1 was salvaged as a whole and transported to the glass warehouse in Maritime Silk Road Museum in Guangdong Province for excavation. In 2013, the Nanhai No. 1 shipwreck began to be comprehensively excavated, and precious cultural artifacts such as lacquerware, porcelain, and ironware, etc., were found in the shipwreck. The Nanhai No. 1 shipwreck is a bright pearl on the maritime Silk Road, which is of great value to the study of ancient Chinese shipbuilding technology and navigation technology (Yong, 2019).

In the process of excavation and protection of cultural artifacts, biodeterioration is a very serious challenge, which may cause irreversible damage to cultural artifacts. Bacteria, archaea, fungi, and other microorganisms, due to their potential biodeterioration, are a major problem in the process of cultural heritage protection (Sterflinger and Piñar, 2013). There are many forms of cultural artifacts, including stoneware, pottery, ironware, porcelain, lacquerware, handicrafts, calligraphy, and painting, etc. Among them, wooden cultural artifacts exist in many cultural sites, usually in the form of houses, tombs, ships, decorations, and so on (Bjordal et al., 1999; Capretti et al., 2008; Liping et al., 2012). Previous studies have shown that wooden artifacts are excellent organic substrates for fungal growth (Gao et al., 2017). If wooden artifacts are separated from the original storage environment and the environmental conditions become appropriate, wooden artifacts can well support the growth of microorganisms (Gengling et al., 2006). The growth of fungi will change the chemical and physical properties of the material until the wooden artifacts are destroyed (El-Gamal et al., 2016).

Compared to other microorganisms, fungi play an important role in the biodegradation of woody cultural relics. The growth environment of fungi is more extensive, and most fungi can grow in lower temperature and humidity (Liu et al., 2018b). In the process of excavation, wooden relics may be contaminated by microorganisms, especially filamentous fungi, which may cause biodeterioration (Irbe et al., 2012; Ortiz et al., 2014). For example, *Hypochnicium* sp. is the major fungus in the wooden tomb in the “M2” of Dingtao King Mausoleum in Shandong Province (Liu et al., 2017); *Eurotium halophilicum* and *Aspergillus penicillioides* are the major fungi of desk and leather luggage rack in the C7 storage room of Tianjin Museum (Liu et al., 2018b); *Penicillium* sp., *Cladosporium* sp., and *Exophiala* sp. are the major fungi of a canoe in Tang Dynasty of China National Marine Museum (Zhang et al., 2019). In 2015, Liu’s research on the fungi biodeterioration of Nanhai No. 1 found that *Fusarium* sp. was the major fungus on the hull of Nanhai No. 1 shipwreck (Liu et al., 2018a). The ability of fungi to destroy wooden artifacts is determined by a combination of parameters, such as interactions of various enzymes in fungi, interactions with other organisms (such as bacteria), and moisture, temperature, environmental humidity, and so on (Pietikäinen et al., 2004; Setälä and Mclean, 2004; Hunt, 2012). Fungi play a vital role in the decomposition of organic matter, especially cellulose and lignin, and many studies have shown that many fungi can produce hemicellulase, cellulase, and lignin degrading enzyme (Torre and Gomez-Alarcon, 1994; Lynd et al., 2002; Coleman et al., 2007; Chinedu et al., 2008; Sánchez, 2009). The ability of fungi to degrade lignin and cellulose will cause irreversible damage to wooden cultural relics, which must be paid more attention.

When fungal biodeterioration occur, traditional fungal detection and identification generally adopt a method of culture separation and microscopic observation, which can lock the cultivable fungal species within a certain range (Ward et al., 1990). At the same time, fungi that cannot be cultured are often identified by high-throughput sequencing, which has been widely used in clinical medicine and ecological environment research (Shokralla et al., 2012; Goodwin et al., 2016). Therefore, if traditional methods can be combined with high-throughput sequencing, the composition of the microbial community structure can be better characterized (Dissanayake et al., 2018; Jayawardena et al., 2018), so as to better protect and research cultural heritage.

The comprehensive excavation of the Nanhai No. 1 shipwreck began in 2013. As the archeological excavation progressed, we discovered that biological plaques had been generated on the hull being excavated and the sea mud around the hull. At the same time, some of the hull wood stored in a specific solution with Euxyl^®^K100 and EDTA-2Na appeared biological containmination. To this end, we performed exposed and soaked wood and glass warehouse air sampling, using scanning electron microscope (SEM) and optical microscope, high-throughput sequencing, and culture-based methods to conduct a comprehensive analysis of the fungal community. In addition, we also isolated the major fungi, tested their ability to degrade lignin and cellulose, evaluated the antifungal effect of biocides, and measured its growth curve. The results of the above research will provide a reference to protect the Nanhai No. 1 shipwreck and other similar cultural relics in future.

## Materials and Methods

### Description of the Sampling Object

The Nanhai No. 1 shipwreck was preserved in a huge enclosed glass warehouse in the Maritime Silk Road Museum for excavation. It is a Song Dynasty ocean trading merchant ship with an earlier age, a larger hull, and relatively complete preservation. The hull has a residual length of 22.95 meters and a width of 9.85 meters. The annual average temperature in the glass warehouse is 25.6°C and the annual average humidity is 84.1% (Liu et al., 2018a). With the progress of archeological excavation, visible fungal contamination has appeared on the excavated hull and the sea mud around the hull, and at the same time, some of the hull wood stored in a specific solution also had microbial contamination.

### Sample Collection

#### Sampling of Hull and Sea Mud

**FIGURE 1:**
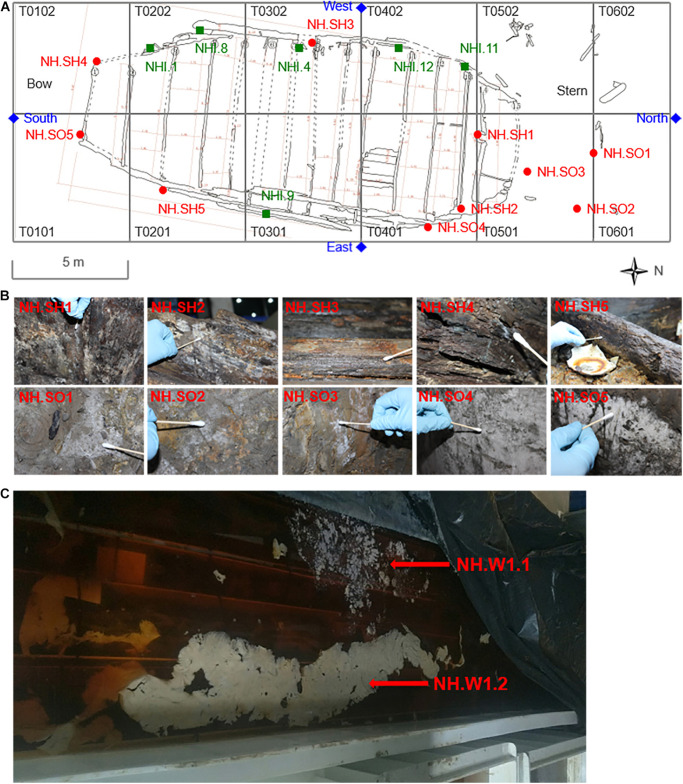
Specific locations and sampling pictures of the hull, sea mud, wood storing water, and air samples in the glass warehouse sampling points of the Nanhai No. 1. **(A)** The T0101–T0602 are different excavation areas in the platform of the shipwreck. The red round dots represent the sampling points of the hull and the sea mud, and the blue diamond marks represent the sampling points of the air in the glass warehouse. The green square marks represent the sampling points of the hull in 2015 (Liu et al.). **(B)** The images of 10 samples that were taken from the hull and sea mud, white biofilm was obviously visible on the surface of the samples. **(C)** The wood storing water samples stored in the specific solution was taken from the water tank beside the excavation site.

The hull and sea mud samples of the Nanhai No. 1 shipwreck were collected at different locations. The sampling time was November 2019. The specific sampling locations are shown in Figure 1A. A total of 10 sites were collected, namely NH.SH1-NH.SH5 and NH.SO1-NH.SO5 (Figure 1B), SH and SO represent the sample from the hull and sea mud, respectively. Sampling was conducted in three steps: First, the fungal contamination was sticked by double-sided carbon conductive adhesive for SEM observation; then, the visible contamination at the same location was inoculated into potato dextrose agar (PDA) medium, which was used for fungal isolation and identification; finally, enough samples were scraped into the EP tube at the same location for total DNA extraction and biodiversity analysis.

#### Collection of Hull Wood Storing Water Samples

Part of the hull wood was stored in a specific solution for more than 1 year (Figure 1C), 0.7‰ Euxyl^®^ K100 and 10 mmol/L EDTA-2Na were added to the water tank. The combination of EDTA-2Na and Euxyl^®^ K100 can play the role of desulphurization, iron removal and fungal inhibition at the same time (Zhang et al., 2014; Ji et al., 2016). Before storage, these hull woods were thoroughly cleaned, basically removing the visible sea mud on the surface. The sampling time was September 2019. At the time of sampling, the pH in the water tank is 6.83 and the water temperature is 24.5°C. Two 50 ml sterile centrifuge tubes were used to collect 40 ml wood storing water samples, named NH.W1.1 and NH.W1.2, respectively, and take them to the laboratory in an icebox for optical microscopic observation and biodiversity analysis.

#### Collection of Air Samples in the Glass Warehouse

The temperature and humidity of the Nanhai No. 1 excavation site were 21.6°C and 74.3% in November 2019. The glass warehouse is an enclosed space with a total volume of about 8,738 square meters. We collected microorganisms in the glass warehouse air at four points (Figure 1A): North, South, East, and West of the hull to analyze the fungal community in the excavation environment of the Nanhai No. 1. Zr-2050 air sampler (junray, China) was used for air sampling, with a sampling flow rate of 100 L/min and a working time of 2 min. Two replicates of 200 L air were collected at each location using the PDA medium. These media were then brought back to the laboratory for fungal isolation and identification. Finally, the fungal concentration will be calculated in colony forming units per cubic meter (CFU/m^3^), and draw the fungi distribution map.

### SEM and Optical Microscope Observation

The hull and sea mud samples obtained with double-sided carbon conductive adhesive were glued to the sample cups and sprayed with gold. The gold-plated samples were observed with SEM (FEI Quanta 200, United States), and images were obtained at a magnification of 1 k× to 5 k×. The same method was used to observe the morphology of mycelia and spores of dominant fungi. The optical microscope (Nikon E200, Japan) was used to observe the diluted wood storing water samples and record the microbial morphology under 40 times microscope.

### Total DNA Extractions and High-Throughput Sequencing

We used the DNeasy PowerSoil Kit (QIAGEN, Germany) to extract the total DNA of 10 samples of the hull and sea mud (NH.SH1-NH.SH5 and NH.SO1-NH.SO5). At the same time, We used the DNeasy PowerWater Kit (QIAGEN, Germany) to extract the total DNA of two wood storing water samples (NH.W1.1 and NH.W1.2). The total DNA was sent to Novogene Genome Sequencing Company. The ITS1-5F region was sequenced by IonS5TMXL platform, and the fungal community in the samples was analyzed using the sequencing results.

### Isolation and Identification of Dominant Fungi

We used PDA medium to culture and isolate fungi. We wrapped the collected PDA medium back to the laboratory. At the same time, the wood storing water samples were diluted in gradient, and 10^–2^ and 10^–3^ dilutions were applied to the PDA medium. The PDA mediums were cultured at 28°C for 3–7 days. Firstly, the strains in the glass warehouse air samples were classified and counted according to morphology. Then the fungi with significantly different morphology in each medium were isolated and purified. After 1–3 times of separation, pure isolates were obtained for DNA extraction and strain identification. The T5 Direct PCR Mix kit (TSINGKE, China) was used for DNA extraction and PCR amplification of the dominant fungi according to the manufacturer’s protocol. The ITS1-5.8srRNA-ITS2 gene of the fungus was amplified with ITS1/ITS4 primers (White et al., 1990). The PCR system and PCR conditions are detailed in the manual of the kit. The unpurified PCR products were sent to GENEWIZ (Beijing, China) for sequencing and then the sequence homology of the amplified fragments was analyzed by NCBI. After isolation and identification of fungi, we deposited the main pure cultures of fungi. The mycelia and spores of fungi were collected with ddH_2_O, and the 1 ml collection was deposited in 500 μL 60% glycerin and frozen at −80°C.

### Ligninolytic and Cellulolytic Enzymatic Activity of Dominant Fungi

In order to test the activity of ligninolytic enzymes and cellulolytic enzymes, potato dextrose agar guaiacol (PDA-guaiacol) medium (Viswanath et al., 2008) and Carboxymethylcellulose sodium (CMC) solid medium (Kasana et al., 2008) were prepared, respectively. The iodine-potassium iodide solution consisted of 1.0 *g* KI and 0.5 *g* iodine in 150 ml distilled water for the reaction of the CMC solid medium for 5 min in the dark. The dominant fungi strains were inoculated into the above two mediums and cultured at 28°C for 7 days and 9 days, respectively, to evaluate the degradation ability of lignin and cellulose. We repeated the experiment three times for each medium. In the PDA guaiacol medium, ligninolytic enzyme can catalyze guaiacol and then a brown-red area was formed in the center of the medium. The ability of fungi to degrade lignin is proportional to the size and depth of the red-brown area (Viswanath et al., 2008; Qing-Fang and Zong-Lian, 2014). In the CMC solid medium, cellulose is the only carbon source. The size of the transparent circle is observed after the reaction with the iodine-potassium iodide solution. The size of the transparent circle is directly proportional to the activity of cellulolytic enzymes (Kasana et al., 2008).

### The Susceptibility of Dominant Fungi to Biocides

Biocides can effectively inhibit the growth of fungi. For example, biotin T, and isothiazolinone have been widely used in the protection of cultural heritage (Polo et al., 2010; de los Ríos et al., 2012; Junqian et al., 2017). The biocides we selected are Preventol^®^ D7, Preventol^®^ BIT 20N, Preventol^®^ P91, Euxyl^®^ K100, Nystatin, Voriconazole, and Amphotericin B (Supplementary Table 1). The first four are isothiazolinone derivatives (Ji et al., 2016). Among them, Euxyl^®^ K100 with a concentration of 0.5% has been used to inhibit the fungal biodeterioration of the hull wood of the Nanhai No. 1. We used the paper diffusion method to determine the sensitivity of dominant fungi to biocides. The fungi were evenly inoculated on PDA medium, and then 5 pieces of filter paper with a diameter of 7 mm were placed, and different biocides and control solutions were applied, respectively. After culturing at 28°C for 3 days, observe the size of the inhibition zone. The larger the diameter of inhibition zone, the higher the sensitivity of the dominant fungi to biocides (Zhang et al., 2019). Three repeat tests are required for all biocide tests.

### Growth Curve of Dominant Fungi

In order to determine the growth activity of the dominant fungi and observe the growth status of the dominant fungi, we drew the growth curve of the dominant fungi. We prepared potato dextrose (PD) liquid medium to culture the dominant fungi. First, the spores of the dominant fungi were collected and the number of spores was calculated. Then took about 8 × 10^4^ spores and added them to 100 ml PD liquid medium, 33 bottles in total, and cultured them at 28°C with 150 rpm. Three bottles were taken every 24 h to determine the dry weight of mycelium, and three bottles were parallel samples, a total of 11 days were measured. The collected mycelium suspension was freeze-dried continuously for 42 h with a freeze-drying machine (SP Scientific virtis, United States), and then the mycelium dry weight was weighed. Finally, the growth curve of dominant fungi was drawn with the average dry weight of mycelium as the ordinate and the days of cultivation as the abscissa.

## Results

### SEM and Optical Microscope Observation

**FIGURE 2:**
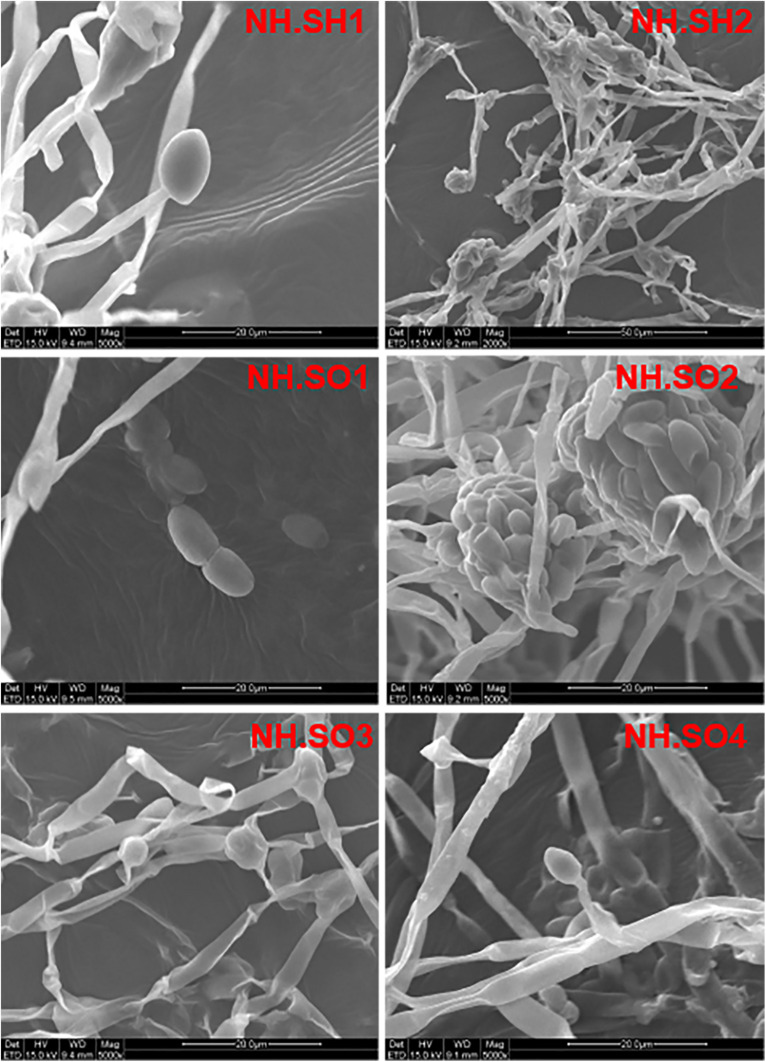
Scanning electron micrograph. The diameter of fungal mycelia is 2–4 μm, the spores are oval and kidney-shaped, and the number of conidial fructification and conidia are large. The scale is 20–50 μm.

In order to study the microbial biodeterioration on the hull and sea mud of the Nanhai No. 1, six samples (NH.SH1-NH.SH2 and NH.SO1-NH.SO4) of double-sided carbon conductive adhesive were observed using SEM. The results showed that there are filamentous fungi on the surface of all the samples, including mycelia, spores, and conidial fructification (Figure 2). The abundance of mycelia and spores showed that significant fungal colonization appeared on the surface of the hull and sea mud. The optical microscope was used to observe the wood storing water samples. Under a 40x microscope, many fungal-like microorganisms can be seen (Supplementary Figure 1), indicating obvious fungal colonization in the wood storing water samples.

### Fungal Community Analysis by High-Throughput Sequencing

**FIGURE 3:**
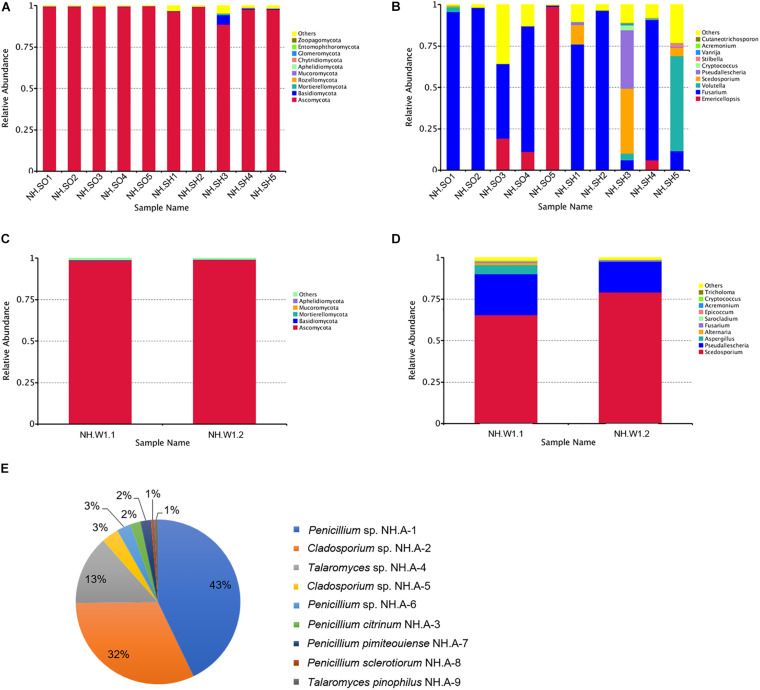
Relative abundances of fungal communities in the hull, sea mud, wood storing water samples, and air in the glass warehouse. The relative abundance is shown as a percentage. Fungal phylum or genera are colored according to the legend on the right. **(A)** Relative abundances of the fungal phyla among hull and sea mud samples; **(B)** Relative abundances of the fungal genus among hull and sea mud samples. **(C)** Relative abundances of the fungal phyla among wood storing water samples; and **(D)** Relative abundances of the fungal genus among wood storing water samples. **(E)** Fungal distribution map of the air in the glass warehouse.

High-throughput sequencing of the ITS1-5F region was performed on the IonS5TMXL platform to analyze the diversity of fungal communities in hull, sea mud, and wood storing water samples. Figures 3A,B show the distribution of dominant fungi in 10 samples from the hull and sea mud. Figure 3A represents the distribution of the fungal phylum in 10 samples. The dominant phylum in the 10 samples was Ascomycota. Ascomycota presents in all 10 samples, accounting for 88.75%∼99.83% of the community, with an average relative abundance of 97.76%. Basidiomycota also exists in 10 samples, accounting for 0.13%∼0.69% of the fungal community, with an average relative abundance of 0.84%. Rozellomycota, Mortierellomycota, and Mucoromycota are only present in some samples. Figure 3B and Supplementary Table 2 represent the distribution of the fungal genus in 10 samples. Of the 10 most abundant fungal groups, *Fusarium* sp., *Emericellopsis* sp., *Volutella* sp., *Scedosporium* sp., and *Pseudallescheria* sp. account for a large proportion. *Fusarium* sp. is the most abundant genus among 7 samples (NH.SH1, NH.SH2, NH.SH4, and NH.SO1-NH.SO4), accounting for 44.96%∼97.99% of the entire community, with an average relative abundance of 81.62%. In NH.SO5 sample, the most abundant is *Emericellopsis* sp., accounting for 98.73%. In NH.SH3 sample, the most abundant is *Scedosporium* sp. and *Pseudallescheria* sp., accounting for 39.17% and 35.45%, respectively. In NH.SH5 sample, the most abundant is *Volutella* sp., accounting for 57.44%. Other fungal genera account for only a small portion. The above results indicate that there are some similarities and differences in the fungal communities of different sampling sites, and *Fusarium* sp. is still widespread on the surface of the Nanhai No. 1 hull and sea mud.

Figures 3C,D show the distribution of dominant fungi in two wood storing water samples. At the phylum level (Figure 3C), Ascomycota accounted for a large proportion, with an average relative abundance of 98.73%. At the genus level (Figure 3D), the dominant genus in samples is *Scedosporium* sp., accounting for 65.45% and 79.1% of NH.W1.1 and NH.W1.2, respectively, with an average relative abundance of 72.28%. *Pseudallescheria* sp., *Aspergillus* sp., *Alternaria* sp., and *Fusarium* sp. also exist in two wood storing water samples, with an average relative abundance of 21.62%, 2.84%, 0.66%, and 0.62%, respectively. The above results indicate that the fungal communities at NH.W1.1 and NH.W1.2 overlap to a certain extent, and the major fungus in the wood water storing samples is *Scedosporium* sp.

### Isolation and Identification of Dominant Fungi by Culture-Dependent Methods

The hull and sea mud samples inoculated in PDA medium were cultured at 28°C for 5 days, all produced the same pure white (RAL 9010) aerial mycelia, the back was cream yellow (RAL 9001), the mycelium was flocculent, vigorous growth, and can produce pigments to make the medium strawberry red (RAL 3018). A filamentous fungus was isolated, and the ITS gene region was amplified and sequenced. The analysis results showed that it has a 100% sequence similarity with *F. solani* (NK-NH1), and the identification results are shown in Table 1. Based on the results of high-throughput sequencing, molecular identification, and morphological characteristics of the culture, we conclude that *F. solani* is the main *Fusarium* sp. on the hull and sea mud of the Nanhai No. 1 (Liu et al., 2018a).

**TABLE 1:** Molecular identification of strains isolated from the hull, sea mud, water, and air.

Fungi	Closet relative strain	Phylum	Similarity (%)	Accession number	Source
NK-NH1	*Fusarium solani*	Ascomycota	100%	KY410238.1	Hull and sea mud
NH.W1-1	*Penicillium* sp.	Ascomycota	100%	GU212865.1	Water
NH.W1-2	*Penicillium citrinum*	Ascomycota	100%	MN398977.1	Water
NH.W1-3	*Scedosporium apiospermum*	Ascomycota	100%	FJ713053.1	Water
NH.W1-4	*Fusarium solani*	Ascomycota	100%	MN066126.1	Water
NH.W1-5	*Cladosporium* sp.	Deuteromycotina	99%	MN265985.1	Water
NH.A-1	*Penicillium* sp.	Ascomycota	100%	MN518406.1	Air
NH.A-2	*Cladosporium* sp.	Deuteromycotina	100%	MH985344.1	Air
NH.A-3	*Penicillium citrinum*	Ascomycota	100%	MN736554.1	Air
NH.A-4	*Talaromyces* sp.	Ascomycota	100%	MH935987.1	Air
NH.A-5	*Cladosporium* sp.	Deuteromycotina	99%	KY621330.1	Air
NH.A-6	*Penicillium* sp.	Ascomycota	100%	MN640089.1	Air
NH.A-7	*Penicillium pimiteouiense*	Ascomycota	100%	KC344973.1	Air
NH.A-8	*Penicillium sclerotiorum*	Ascomycota	100%	MG827186.1	Air
NH.A-9	*Talaromyces pinophilus*	Ascomycota	100%	MF686811.1	Air

**FIGURE 4:**
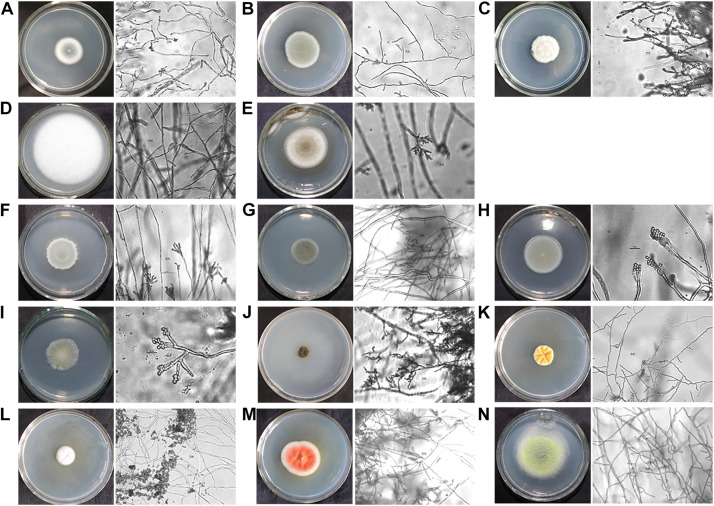
Single colony morphology and micromorphology of fungal isolates from wood storing water samples and air in the glass warehouse. The fungi were inoculated onto the PDA medium and incubated at 28°C for 3 days. The scale is 10 μm. **(A)** *Penicillium* sp. (NH.W1-1). **(B)** *P. citrinum* (NH.W1-2). **(C)** *S. apiospermum* (NH.W1-3). **(D)** *F. solani* (NH.W1-4). **(E)** *Cladosporium* sp. (NH.W1-5). **(F)** *Penicillium* sp. (NH.A-1). **(G)** *Cladosporium* sp. (NH.A-2). **(H)** *P. citrinum* (NH.A-3). **(I)** *Talaromyces* sp. (NH.A-4). **(J)** *Cladosporium* sp. (NH.A-5). **(K)** *Penicillium* sp. (NH.A-6). **(L)** *P. pimiteouiense* (NH.A-7). **(M)** *P. sclerotiorum* (NH.A-8). **(N)** *T. pinophilus* (NH.A-9).

**FIGURE 5:**
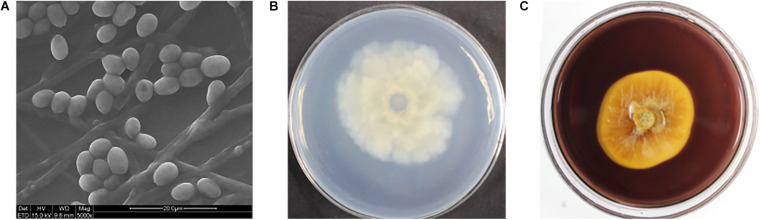
**(A)** Scanning electron micrograph of *S. apiospermum* (NH.W1-3). The diameter of the fungus mycelia is 1.5–4 μm, and the spores are oval. The scale is 20–50 μm. **(B)** Colony appearances of *S. apiospermum* (NH.W1-3) grown on the PDA-guaiacol medium for 7 days. (back of medium; **(C)** Colony appearances of *S. apiospermum* (NH.W1-3) grown on the CMC medium for 9 days. (front of medium).

The hull wood storing water samples were diluted and coated on the PDA medium. After cultured at 28°C for 5 days, different fungal colonies were isolated and purified. Five different fungi were obtained, which were NH.W1-1 to NH.W1-5. The obtained pure isolates were subjected to amplification and sequencing of the ITS gene region. The analysis results are shown in Table 1. The morphology of the mycelia and spores of these five fungi were observed and recorded with optical microscope (Figures 4A–E). For further research, we observed the mycelia and spores of *S. apiospermum* (NH.W1-3) using SEM (Figure 5A). Combined with the high-throughput sequencing, molecular identification results, and the morphological characteristics of the cultures, we conclude that *S. apiospermum* (NH.W1-3) is the major fungus of wood storing water samples.

Air samples in the glass warehouse were collected at four locations in the North, South, East, and West of the hull. 200 L of air was taken in the PDA medium. After cultured at 28°C for 5 days, the fungal strains were classified and counted, and then purified and identified. We isolated nine fungi (Table 1), numbered NH.A-1 to NH.A-9. Morphology of the mycelia and spores of these nine fungi were observed and recorded with optical microscope (Figures 4F–N). The fungal concentration was calculated as colony forming units per cubic meter (CFU/m^3^). At the four points of North, South, East, and West of the hull, the fungal load was 235, 160, 165, and 175 CFU/m^3^, respectively, with an average load of 183.75 CFU/m^3^. The fungi isolated from the air in the glass warehouse are summarized in Figure 3E after classification and counting. In the glass warehouse air samples, the isolated fungi include *Penicillium* sp. (NH.A-1 and NH.A-6), *Penicillium citrinum* (NH.A-3), *Penicillium pimiteouiense* (NH.A-7), *Penicillium sclerotiorum* (NH.A-8), *Talaromyces* sp. (NH.A-4), *Talaromyces pinophilus* (NH.A-9), and *Cladosporium* sp. (NH.A-2 and NH.A-5). Four species belonging to the genus *Penicillium* account for 51% of the total. Two species belonging to the genus *Cladosporium* account for 45% of the total. Two species belonging to the genus *Talaromyces* account for 4% of the total. Therefore, the strains isolated from the air in the glass warehouse are mainly *Penicillium* sp. and *Cladosporium* sp.

### Ligninolytic and Cellulolytic Enzymatic Activity of *S. apiospermum* (NH.W1-3)

As *S. apiospermum* (NH.W1-3) is the most important fungus in wood storing water samples, we speculate that *S. apiospermum* (NH.W1-3) may produce enzymes that can decompose lignin or cellulose, and thus cause degradation of water-saturated wood. We inoculated the strain *S. apiospermum* (NH.W1-3) on the PDA-guaiacol medium and CMC solid medium, and cultured at 28°C for 7–9 days. It was found that there was no brown-red area on the PDA-guaiacol medium (Figure 5B), hence *S. apiospermum* (NH.W1-3) did not have lignocellulolytic enzyme activity. At the same time, an obvious transparent circle appeared on the CMC solid medium (Figure 5C), indicating that the strain can utilize cellulose. In summary, the *S. apiospermum* (NH.W1-3) strain may have certain wood biodegradation ability, but the reason for its large presence in wood storing water samples needs further investigation.

### The Susceptibility of Dominant Fungi to Biocides

**FIGURE 6:**
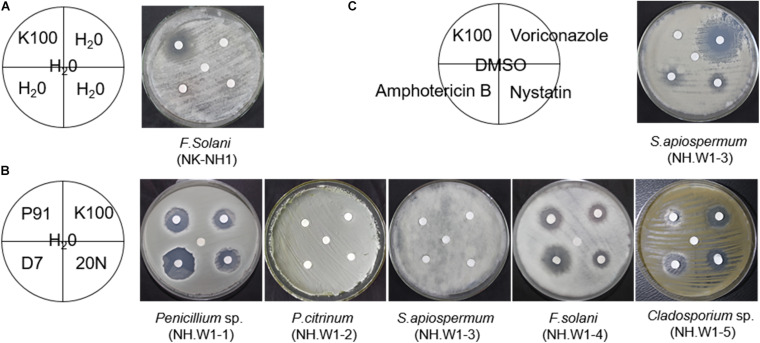
Sensitivity of fungal isolates to biocides. The inhibition zone shows the effectiveness of biocides against fungal strains. All fungal isolates were cultured at 28°C for 3 day. **(A)** *F. solani* (NK-NH1). K100 used. **(B)** *Penicillium* sp. (NH.W1-1), *P. citrinum* (NH.W1-2), *S. apiospermum* (NH.W1-3), *F. solani* (NH.W1-4), and *Cladosporium* sp. (NH.W1-5). P91, K100, D7, and 20N used. **(C)** *S. apiospermum* (NH.W1-3). Amphotericin B, Voriconazole, K100, and Nystatin used.

Six fungi (NK-NH1 and NH.W1-1 to NH.W1-5) were purified from hull, sea mud, and wood storing water samples. The concentration of Euxyl^®^ K100 applied to the hull and wood storing water samples on site were 0.5% and 0.7‰, respectively. We used the paper diffusion method to determine the sensitivity of fungi to four biocides, i.e., Preventol^®^ D7, Preventol^®^ BIT 20N, Preventol^®^ P91, and Euxyl^®^ K100. The concentration of the isothiazolinone derivatives biocide was 0.5%, which was lower than the manufacturer’s recommended concentration of 2% in the manual. Different biocides have different inhibitory effects on different fungi. The *F. solani* (NK-NH1) isolated from the hull and sea mud was the same as the fungus isolated by Liu 2 years ago (Liu et al., 2018a). The results showed that when the concentration of Euxyl^®^ K100 was 0.5% (Figure 6A), it still had an inhibitory effect on it, indicating that *F. solani* did not develop significant resistance to Euxyl^®^ K100. At the same time, among the five fungi isolated from wood storing water samples, the isothiazolinone biocides had a better killing effect on *Penicillium* sp. (NH.W1-1), *F. solani* (NH.W1-4), and *Cladosporium* sp. (NH.W1-5), but on *P. citrinum* (NH.W1-2), and *S. apiospermum* (NH.W1-3) has almost no fungistatic effect (Figure 6B). Among the four biocides, D7 and P91 have a better fungistatic effect, while Euxyl^®^K100 has the least obvious effect. In addition, we also used the commonly used laboratory and clinical biocides for the treatment of *S. apiospermum* (NH.W1-3), including nystatin, voriconazole, and amphotericin B, to determine the biocide sensitivity of *S. apiospermum* (NH.W1-3; Figure 6C). According to many preliminary experiments, we selected Euxyl^®^ K100 concentration of 0.5%, nystatin concentration of 5 mg/mL, voriconazole concentration of 200 μg/mL, and amphotericin B concentration of 5 mg/mL. We found that *S. apiospermum* (NH.W1-3) has the highest sensitivity to voriconazole, and its sensitivity to nystatin and amphotericin B has certain manifestations at higher concentrations.

### Growth Curve of *F. solani* (NK-NH1)

In order to observe the growth of the dominant fungi *F. solani* (NK-NH1), we drew its growth curve (Supplementary Figure 2). From the figure, we can see that the growth of *F. solani* (NK-NH1) is basically in a rising state before the eighth day of culture, and the growth of *F. solani* (NK-NH1) is in a state of decline from the ninth day. The results showed that the growth of *F. solani* (NK-NH1) is more vigorous under suitable growth conditions.

## Discussion

The purpose of this regular inspection and sampling of the Nanhai No. 1 is to detect and analyze the fungal community in the preservation environment of the Nanhai No. 1 in order to provide information for future conservation work. A total of three types of samples were collected: hull and sea mud, wood storing water samples, and air in the glass warehouse.

The hull and sea mud samples were observed by SEM, and the white plaque was determined to be a large-scale colonization of fungi, which may cause damage to the hull. Among the hull and sea mud samples, Ascomycota prevailed at the phylum level. Ascomycota was present in all samples with an average relative abundance of 97.76%. Basidiomycota was also present in all samples with an average relative abundance of 0.84%. Rozellomycota, Mortierellomycota, and Mucoromycota are only present in some samples. Wood is rich in nutrients, and some fungal strains can degrade wood (Boddy and Watkinson, 1995; Boddy, 2001; Heilmann-Clausen and Christensen, 2005). Wood-decay fungi refer to fungi that grow on wooden substrates, of which about 98% are Ascomycota and Basidiomycota (James et al., 2006). Ascomycota, Basidiomycota, and Rozellomycota are often the main contributors to the biodeterioration of woody artifacts (Irbe et al., 2012; Ortiz et al., 2014; Liu et al., 2018a; Zhang et al., 2019). At the genus level, *Fusarium* sp., *Emericellopsis* sp., *Volutella* sp., *Scedosporium* sp., and *Pseudallescheria* sp. are dominant, accounting for 59.00%, 13.58%, 6.52%, 5.64%, and 3.78%, respectively, while other fungal genera accounted for only a small portion. Compared with Liu’s (Liu et al., 2018a) investigation on the fungal biodeterioration in the hull of the Nanhai No. 1 in 2015, we found that Ascomycota was still dominant at the phylum level, but the average share decreased slightly, while the share of Basidiomycota increased slightly. At the genus level (Supplementary Figure 3), there are many other genera of fungi, such as *Emericellopsis* sp., *Pseudallescheria* sp., *Scedosporium* sp., and *Volutella* sp., which have not appeared before. It is suggested that the fungal community on the part of hull surface may have changed. *Scedosporium* sp. and *Pseudallescheria* sp. also exist in the wood storing water samples and are the main fungi in the wood storing water samples. But overall, the main biodeterioration agent is *Fusarium* sp., which belongs to Ascomycota. *Fusarium* is widely distributed in soil, animal, and plant organisms, and some can live in extreme environments. *Fusarium* sp. can degrade plant debris and has high lignocellulose decomposition activity (Rodriguez et al., 1996; Obruca et al., 2012; Daâssi et al., 2016). The predominant fungus isolated from the hull and sea mud was *F. solani* (NK-NH1), which is similar to the finding of Liu et al. (2018a). *F. solani* (NK-NH1) can colonize on the hull, which might due to the transfer of the hull from the seabed environment to the storage environment with high oxygen and high humidity. The abundant nutrition and suitable growth environment in the hull wood make it reproduce in large areas. Therefore, *F. solani* (NK-NH1) should be regarded as the greatest threat to the hull of the Nanhai No. 1. The concentration of Euxyl^®^K100 sprayed on the hull on site was 0.5%. We used the paper diffusion method to determine the sensitivity of *F. solani* (NK-NH1) to 0.5% of Euxyl^®^ K100. The results showed that Euxyl^®^ K100 still inhibited it, indicating that *F. solani* (NK-NH1) did not produce obvious drug resistance.

Wood storing water samples were observed by optical microscope to get many fungal-like microorganisms, indicating obvious fungal colonization in the wood storing water samples. High-throughput sequencing showed the dominant phylum level in the wood water samples was Ascomycota, which accounted for 98.73% of the community, and the dominant genus level was *Scedosporium* sp., which accounted for 72.28% of the community. Some isolates of *Scedosporium* species, such as *S. apiospermum* and *S. boydii*, are the main pathogens of human beings, which can cause organ failures, the most common is lung infections (Lackner et al., 2014; Michaela et al., 2014). The *S. dehoogii* and *S. minutispora* are mainly isolated from the environment, and few clinical cases have been reported (Cortez et al., 2008; Gilgado et al., 2008; Kaltseis et al., 2009). Five fungi isolated from wood storing water samples were *Penicillium* sp. (NH.W1-1), *P. citrinum* (NH.W1-2), *Scedosporium apiospermum* (NH.W1-3), *Fusarium solani* (NH.W1-4), and *Cladosporium* sp. (NH.W1-5). Combined with the high-throughput sequencing results, *S. apiospermum* (NH.W1-3) is the most important fungus in wood storing water samples. *S. apiospermum* (Sexual period is *Pseudallescheria boydii*) is a human pathogen that can cause organ infections and is more susceptible to infection in immunodeficient patients. The fungus is mainly distributed in temperate regions and can tolerate extreme environments such as high salt and low oxygen. At the same time, the fungus can be resistant to a variety of antifungal drugs, so its treatment is usually very difficult (Bosma et al., 2010; Chaveiro et al., 2010). In the environment, it is a kind of eutrophic fungus, which prefers fertilizer-rich, polluted, and human-affected areas (Fisher et al., 1982; Guarro et al., 2006; Hoog et al., 2010; Rougeron et al., 2015), and can also grow in submerged wood in estuaria (Kirk, 1967) and marine soil (Dabrowa et al., 1964). These five fungi were tested for biocides sensitivity. It was found that the isothiazolinone biocides had a better killing effect on *Penicillium* sp. (NH.W1-1), *F. solani* (NH.W1-4), and *Cladosporium* sp. (NH.W1-5), but had little effect on *P. citrinum* (NH.W1-2), and *S. apiospermum* (NH.W1-3). Among the four isothiazolinone biocides, D7 and P91 have a better antifungal effect, while Euxyl^®^ K100 has the least obvious effect. Therefore, the biocide Euxyl^®^ K100 currently used on site has no antifungal effect on *S. apiospermum* (NH.W1-3). Amphotericin B and voriconazole are usually used to treat the infection caused by *S. apiospermum* (Nomdediu et al., 1993; Girmenia et al., 1998; Nulens et al., 2003). Hence, we used the appropriate concentration of amphotericin B and voriconazole to carry out the sensitivity test, and found that voriconazole had a good effect. Our culture analysis showed that *S. apiospermum* (NH.W1-3) does not has lignocellulolytic enzyme activity but can metabolize cellulose. Therefore, the *S. apiospermum* (NH.W1-3) strain might have the ability to biodegrade wood, but the reason for its large presence in wood storing water samples needs further investigation. It is speculated that the possible reasons for its existence in wood storing water samples are as follows:

(I) *S. apiospermum* (NH.W1-3) has a wide survival range and can grow normally in a hypoxic environment. The pH value in the tank is 6.83, and the water temperature is 24.5°C, which is within the environmental range suitable for the fungus to grow (Rougeron et al., 2015).
(II) The fungus likes to grow in a nutrient-rich environment, and the wood is rich in nutrients, so it can grow favorably in the water sample.
(III) The fungus is resistant to many antifungal drugs. It can grow in a pool containing Euxyl^®^ K100 at a concentration of 0.7‰, indicating that it is resistant to isothiazolinone drugs.

Air samples in the glass warehouse were collected at four locations in the North, South, East, and West of the hull, the average fungal load was 183.75 CFU/m^3^. We isolated nine fungi, numbered NH.A-1 to NH.A-9, of which *Penicillium* sp. accounted for 51%, *Cladosporium* sp. accounted for 45%, and *Talaromyces* sp. accounted for 4% of the total number. Therefore, *Penicillium* sp. and *Cladosporium* sp. were abundant in the air in the glass warehouse. Studies have shown that *Penicillium* sp. and *Cladosporium* sp. are dominant in the air of the urban agglomeration of Guangdong Province (Zheng et al., 2009). Fungi can exist in the air in the form of spores and hyphae, which will cause colonization on the surface of cultural artifacts (Zielinska-Jankiewicz et al., 2008; Abdel-Kareem, 2010; Skora et al., 2015). For example, the major fungi in the air of the Tianjin Museum are *Penicillium* sp. and *Cladosporium* sp. (Zhang et al., 2019). *Cladosporium* sp. was detected in the air of an art museum in Tokyo (Abe, 2010). The prevailing fungal genera *Cladosporium* sp. and *Penicillium* sp. were isolated from archives of air samples in Argentina and Republic of Cuba (Borrego et al., 2012). *Penicillium* sp. and *Cladosporium* sp. can secrete a variety of lignin and cellulose-degrading enzymes, and can be colonized on wooden artifacts to cause damage (Singh and Flegel, 1986). Therefore, *Penicillium* sp. and *Cladosporium* sp. should also be considered as potential hazards in the preservation of cultural artifacts in the Nanhai No. 1.

In view of the above, we usually use physical measures such as environmental parameter control and chemical measures such as biocides to protect cultural artifacts. Among them, physical measures such as environmental parameter control is the most widely used. Environmental parameters mainly include temperature and humidity, etc. Biodeterioration agent are usually suitable for growth in warm, humid, and oxygen-rich environments. The temperature at the Nanhai No. 1 excavation site was 21.6°C and the humidity was 74.3%, which greatly accelerated the growth of fungi and their decomposition of wooden artifacts. The optimal humidity range for fungi is 40–80% (Blanchette, 2000). During the archeological excavation of the Nanhai No. 1, we continued to spray distilled water to maintain the hull moisture, which may directly provide the humidity for fungal growth. Therefore, the humidity control should be adjusted to be lower than the appropriate value for the growth of fungi. In addition, archeologists may bring in some biodeterioration agent from the outside when they enter the museum for work, so people’s access and frequency must be strictly controlled. The water for cultural artifacts should be sterile distilled water or double-distilled water (ddH_2_O), and the water needs to be changed regularly to prevent the accumulation of nutrients. The use of biocides is one of the important methods to control biological contamination (Polo et al., 2010; de los Ríos et al., 2012). However, the abuse of biocides may cause microbes to develop drug resistance, and some biocides may cause harm to archeologists and the environment (Kathiravan et al., 2012; Coutinho et al., 2016; Luz et al., 2018). Therefore, the concentration of biocides used at present should not be too high, and it is necessary to assist other physical methods to remove biodeterioration agent. At the same time, environmental biocides for dominant fungi need to be found and developed. In addition to controlling environmental parameters and using biocides, we should also regularly monitor fungal biodeterioration. Through high-throughput sequencing and culture methods to analyze the fungal community in an all-round way, in order to evaluate whether the fungal community changes dynamically, so as to better protect the Nanhai No. 1.

## Conclusion

Through the analysis of the hull and sea mud, wood storing water samples, and air in the glass warehouse samples, we carried out an overall assessment of the fungal community in the Nanhai No. 1 preservation environment. Fungal colonization occurs on the hull and sea mud, and the main fungal community on the part of hull surface may have changed. But overall, the dominant genus level is *Fusarium*. The dominant fungus isolated was *F. solani* (NK-NH1), which did not develop resistance to the current biocide Euxyl^®^ K100. In wood storing water samples, the dominant genus is *Scedosporium*. Among the five fungi isolated, *S. apiospermum* (NH.W1-3) is the most important biodeterioration agent, and the currently used biocides have no effect on it, but voriconazole has a significant inhibitory effect on it. The biodegradation of *S. apiospermum* (NH.W1-3) on wood remains to be studied. We have speculated several reasons for its existence through data, which needs to be confirmed by experiments in the later stage. The number of *Penicillium* sp. and *Cladosporium* sp. is the largest in the glass warehouse air. *F. solani* (NK-NH1), *S. apiospermum* (NH.W1-3), *Penicillium* sp., and *Cladosporium* sp. should be considered as the existing and potential obstacles of the Nanhai No. 1 cultural artifacts preservation. Through discussions, we will use the combination of controlling environmental parameters, using fungicides and regular monitoring to effectively protect the Nanhai No.1.

## Data Availability Statement

The raw sequencing data could be downloaded at the NCBI Sequence Read Archive (SRA) with the study accession number PRJNA663758.

## Author Contributions

JP conceived and designed the study. YH, XH, YW, and KM performed the experiments. JD, YC, NL, and ZZ provided assistance during the experiments. YH analyzed the data and wrote the manuscript. JP reviewed and edited the manuscript. All authors read and approved the manuscript.

## Conflict of Interest

The authors declare that the research was conducted in the absence of any commercial or financial relationships that could be construed as a potential conflict of interest.
