# Supplementary material for: Fungal Community and Biodeterioration Analysis of Hull Wood and Its Storage Environment of the Nanhai No. 1 Shipwreck
Source: Front Microbiol. 2021 Jan 15;11:609475. doi: 10.3389/fmicb.2020.609475 (PMC7843524; doi:10.3389/fmicb.2020.609475)
Supplement: Supplementary file 1 [file Data_Sheet_1.docx]

Supplementary Material


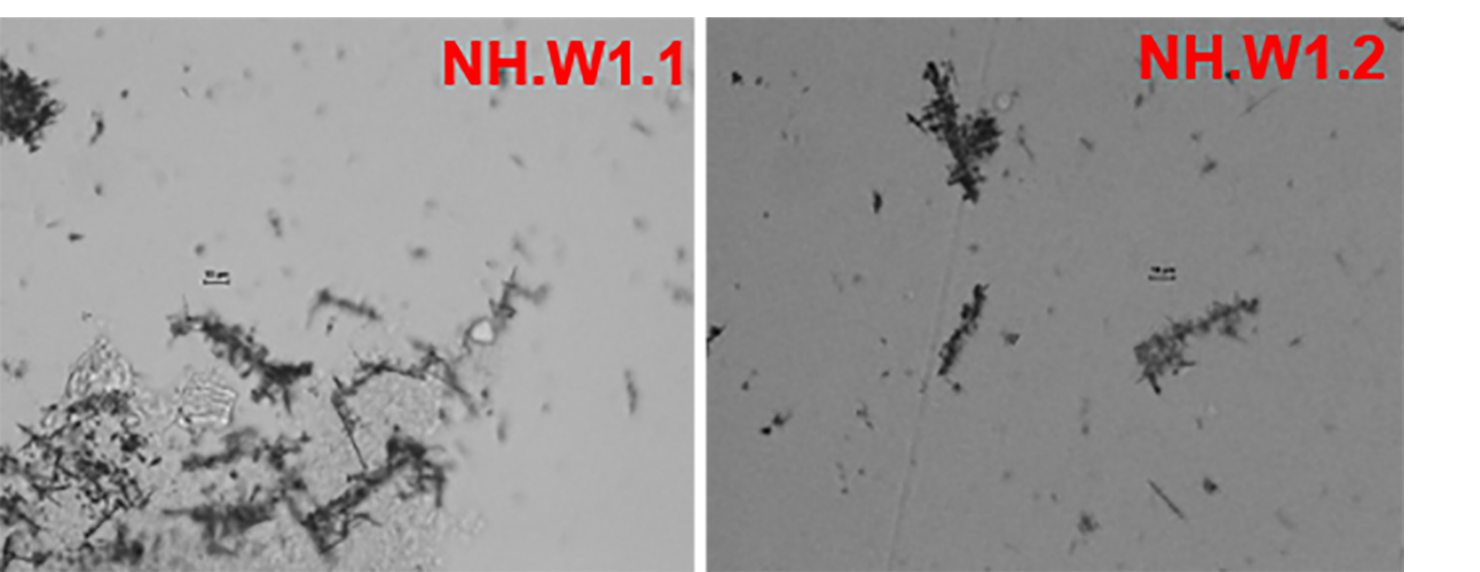


**Supplementary Figure 1.** Optical micrograph. Under the microscope of 40 times, many fungi like microorganisms can be seen. The scale is 10 μm.

**
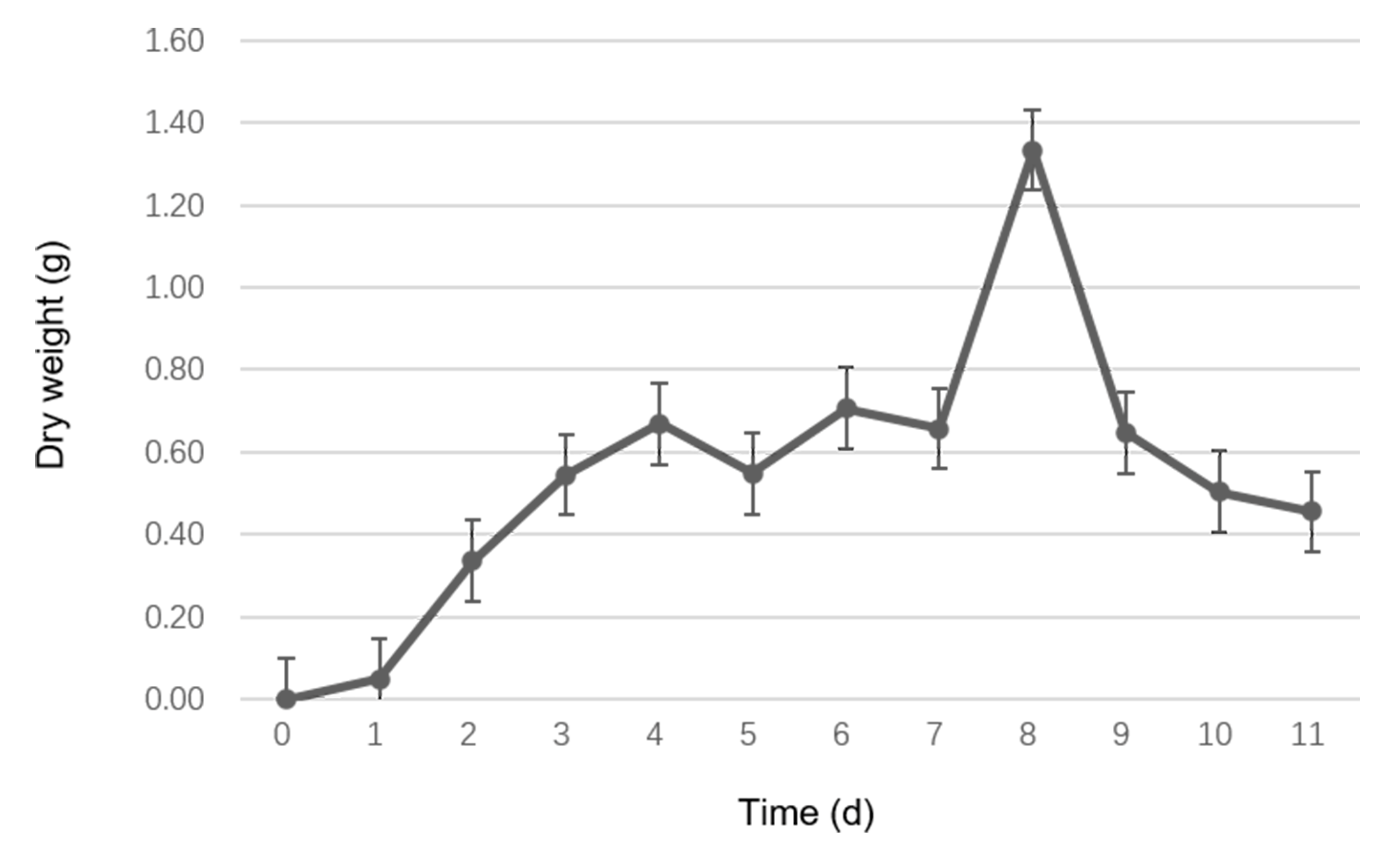
**

**Supplementary Figure 2.** Growth curve of *F. solani* (NK-NH1). The abscissa represents the days of culture, and the ordinate represents the average dry weight of mycelium. The vertical lines indicate standard deviation of three measurements.


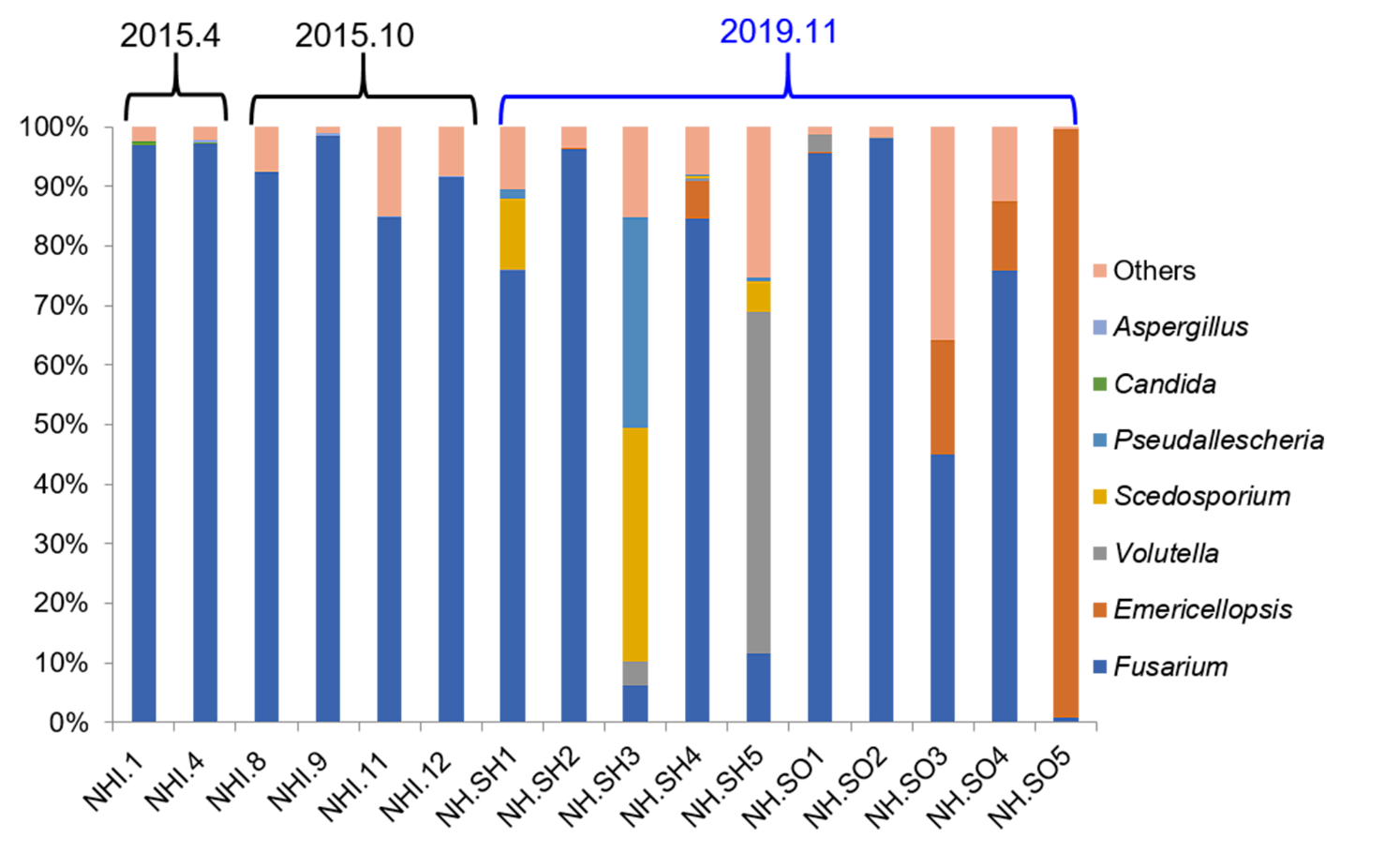


**Supplementary Figure 3.** Through the comparison of the results of Liu et al., it is suggested that the main fungal community at the genus level on the part of hull surface may have changed. But overall, the main disease fungus is *Fusarium* sp.. NHI.1, NHI.4, NHI.8, NHI.9, NHI.11, and NHI.12 are the distribution of fungal communities in April and October 2015 (Liu et al.), NH.SH1-NH.SH5 and NH.SO1-NH.SO5 are the results of this sampling in November 2019.

**Supplementary** **Table 1.** The information of biocides and active compounds.

| Biocides | Active compounds. | Manufactor |
| --- | --- | --- |
| Preventol^®^D7 | Aqueous mixture of multiple isothiazolinones | Lanxess, Germany |
| Preventol^®^BIT 20N | Aqueous mixture of multiple isothiazolinones | Lanxess, Germany |
| Preventol^®^P91 | Bronopol and isothiazolinone | Lanxess, Germany |
| Euxyl^®^ K100 | Benzyl Alcohol and Methylchloroisothiazolinone or Methylisothiazolinone | Schulke, Germany |
| Nystatin | Nystatin | Macklin, Shanghai, China |
| Voriconazole | Voriconazole | Meilunbio^®^, Dalian, China |
| Amphotericin B | Amphotericin B | Meilunbio^®^, Dalian, China |

**Supplementary** **Table 2.** Relative abundance of dominant fungi among hull and sea mud samples at the genus level.

| Dominant Genus（%） | NH.SH1 | NH.SH2 | NH.SH3 | NH.SH4 | NH.SH5 | NH.SO1 | NH.SO2 | NH.SO3 | NH.SO4 | NH.SO5 |
| --- | --- | --- | --- | --- | --- | --- | --- | --- | --- | --- |
| *Fusarium* | 75.89 | 96.32 | 6.13 | 84.63 | 11.57 | 95.66 | 97.99 | 44.96 | 75.90 | 0.9 |
| *Volutella* | 0.27 | 0.01 | 3.98 | 0.57 | 57.44 | 2.89 | 0.01 | 0.03 | 0.01 | 0.01 |
| *Emericellopsis* | 0.02 | 0.14 | 0.05 | 6.18 | 0.01 | 0.07 | 0.15 | 19.31 | 11.15 | 98.73 |
| *Scedosporium* | 11.75 | 0 | 39.17 | 0.46 | 5.03 | 0 | 0 | 0 | 0.01 | 0.01 |
| *Stilbella* | 0.01 | 0 | 0.09 | 0.03 | 2.00 | 0.01 | 0 | 0 | 0 | 0 |
| *Pseudallescheria* | 1.58 | 0 | 35.45 | 0.05 | 0.71 | 0 | 0 | 0 | 0 | 0 |
| *Acremonium* | 0 | 0.01 | 0.09 | 0.03 | 0.23 | 0.48 | 0 | 0 | 0 | 0.09 |
| *Stachybotrys* | 0 | 0.38 | 0 | 0.01 | 0 | 0 | 0 | 0 | 0.01 | 0 |
| *Archaeorhizomyces* | 0.17 | 0 | 0 | 0.13 | 0.30 | 0 | 0 | 0 | 0 | 0 |
| Others | 10.31 | 3.14 | 10.99 | 7.92 | 22.72 | 0.79 | 1.85 | 35.69 | 12.93 | 0.23 |
